# Treatment response of bevacizumab combination chemotherapy in recurrent glioblastoma: A long-term retrospective study in Taiwan

**DOI:** 10.1097/MD.0000000000019226

**Published:** 2020-02-21

**Authors:** Yu-Kai Huang, Ann-Shung Lieu

**Affiliations:** a Graduate Institute of Clinical Medicine, College of Medicine, Kaohsiung Medical University; b Division of Neurosurgery, Department of Surgery, Kaohsiung Medical University Hospital; c Department of Surgery, Kaohsiung Municipal Ta-Tung Hospital, Kaohsiung, Taiwan.

**Keywords:** bevacizumab, carboplatin, chemotherapy, recurrent glioblastoma, vincristine

## Abstract

Treatment options for recurrent glioblastoma are rare, with their response uncertain. This study aimed to determine the response of chemotherapy including bevacizumab in combination with vincristine and carboplatin for glioblastoma at first recurrence in a single-institution cohort.

Clinical data of patients who received chemotherapy including bevacizumab, vincristine, and low-dose carboplatin for recurrent glioblastoma between 2008 and 2014 were analyzed. Differences between those who received combination chemotherapy (chemotherapy-positive) and those who did not (chemotherapy-negative) were estimated by Fisher exact test or Wilcoxon rank-sum test, as appropriate. Survival curves were estimated using the Kaplan–Meier method, and differences between survival curves were estimated by the log-rank test. Univariate analysis of treatment response for all recurrent glioblastoma patients and secondary recurrence patients under different conditions were evaluated using Wilcoxon rank-sum test or the Kruskal–Wallis test.

Although mortality rates were similar between the chemotherapy-negative and chemotherapy-positive groups (26.7% vs 28.6%), median overall survival was significantly longer in the chemotherapy-positive group than the chemotherapy-negative group (*P* = .006). There were no chemotherapy-related serious complications such as gastrointestinal perforation, serious bleeding, or new-onset seizure during chemotherapy, whereas others side effects including proteinuria and hypertension were more common albeit well controlled by medication.

This study revealed combination regimen of bevacizumab, vincristine, and low-dose carboplatin as a potentially effective therapeutic approach in recurrent glioblastoma. More in-depth understanding of the mechanism underlying this combination treatment and potential contribution of alternative genetic therapeutic in recurrent glioblastoma is necessary.

## Abstract 2

Supplemental Digital Content is available in the text

## 1 Introduction

Glioblastoma is the most common malignant central nervous system tumor in adults.^[1,2]^ A population-based registry study in Taiwan reported that 1-year survival rate of glioblastoma was only 50.3%, which was lower at 24.0% at 2 years.^[1]^ Prognosis based on clinicopathologic factors was reported to range from 4.6 to 58.6 months.^[3]^ The poor prognosis associated with glioblastoma from the high propensity for tumor recurrence,^[2,4,5]^ which is usually unavoidable. Nevertheless, no standard treatment has been established for recurrent glioblastoma.^[4,6]^

Glioblastoma is the most vascularized tumor, and angiogenesis is an important component of its pathogenesis.^[7]^ Treatments targeting angiogenesis achieve prolonged survival by inhibiting circulating vascular endothelial growth factor (VEGF), evidenced by a reduction in the size of contrast-enhancing tumors.^[8]^ Therapeutic approaches targeting VEGF expression and its receptor (VEGFR) are the most commonly utilized strategy for glioblastomas.^[9–11]^ The monoclonal antibody bevacizumab, which prevents the interaction between VEGF and VEGFR, is the most common antiangiogenic drug used for newly diagnosed as well as recurrent glioblastomas with acceptable clinical outcomes.^[7,11–13]^

Although single-agent bevacizumab was shown to beneficial by increasing progression-free survival (PFS) in recurrent glioblastoma, similar outcomes were not reported for long-term survival.^[14,15]^ Findings from numerous studies indicate that multiple tumorigenic pathways are involved in glioblastoma development and progression. The effect of single-agent treatment with bevacizumab is thus limited for recurrent glioblastoma treatment, and bevacizumab in combination with other agents is a viable approach.^[16–20]^ Addition of vincristine can achieve inhibition of leukocyte production and maturation, antiangiogenic effects, and antitumor activity in glioblastoma.^[21,22]^ To that end, the efficacy of vincristine, which was reported mainly in newly diagnosed glioblastoma, have not been convincingly shown in recurrent glioblastoma cases, especially in combination with bevacizumab.^[23,24]^ Conversely, carboplatin, another commonly used agent for recurrent glioblastoma, is mainly used in combination with bevacizumab.^[18,19,25,26]^ Although its efficacy in combination with vincristine was documented in previous clinical trials, clinical outcomes of bevacizumab in combination with carboplatin remain unclear.

Adjunctive therapies including alkylating agents such as temozolomide and nitrosoureas with concurrent radiotherapy and surgery led to improvement in glioblastoma outcomes. A previous study reported that median survival was between 12.1 and 14.6 months in glioblastoma patients treated with radiotherapy plus adjuvant temozolomide.^[27]^ Tumor-treating fields, a type of treatment based on low-intensity alternating electric fields, was also shown to contribute to better overall survival (OS) outcomes in recurrent glioblastoma when used with concurrent chemotherapy.^[28,29]^ Reoperation, another viable option for recurrent glioblastoma, might be associated with favorable prognosis.^[30]^

Treatment options for recurrent glioblastoma are rare, and their response remains uncertain. Combination of bevacizumab with valganciclovir in patients with glioblastoma exhibited a trend toward improved survival,^[17]^ whereas bevacizumab in combination with lomustine was shown to be associated with increased OS in patients with recurrent glioblastoma.^[14]^ These results from clinical trials demonstrate that bevacizumab-based combination therapy achieves only modest activity against recurrent tumors.^[14,17]^ While OS and PFS rates have improved with bevacizumab-based combination therapy, there are currently no standard chemotherapy protocols for recurrent glioblastoma. The survival benefit with concurrent use of bevacizumab, vincristine, and carboplatin for recurrent glioblastoma remains unclear. Therefore, we aimed to determine the treatment of bevacizumab/vincristine/carboplatin combination chemotherapy for glioblastoma at first recurrence in a single-institution cohort with a long-term follow-up.

## 2 Methods

### 2.1 Patient selection

This study was approved by the institutional review board of Kaohsiung Medical University hospital (KMUHIRB-G(II)-20170010). In this single-center retrospective study, patients with histologically diagnosed recurrent glioblastoma from 2008 to 2014 were included. Inclusion criteria were defined according to the RENO criteria to determine the recurrence evidence using magnetic resonance imaging (MRI) follow-up. Patients with a mix of different tumor cell origins and completely disabled (Eastern Cooperative Oncology Group performance status grade 4) were excluded. Baseline characteristics and clinicopathologic factors included age, sex, location of recurrence, recurrence type, Karnofsky performance status score,^[31]^ platelet count, hypertension, proteinuria, and treatment outcomes were obtained from hospital medical records.

Patients with first recurrence were scheduled for second surgery and/or considered for bevacizumab/vincristine/carboplatin combination chemotherapy. Indications for surgery or chemotherapy included patient's physical status, economic status, tumor-related mass effect, and presence of brain edema. Treatment flowchart is summarized in APPENDIX A. The combination chemotherapy included 10 mg/kg bevacizumab (avastin), 1 mg/m^2^ vincristine, and 300 mg/m^2^ carboplatin. In this study, patients were categorized into 2 groups: those with at least 1 confirmed recurrence who did not receive any chemotherapy (chemotherapy-negative) and those with recurrence who received bevacizumab/vincristine/carboplatin chemotherapy (chemotherapy-positive). In addition, patients with confirmed secondary recurrent glioblastoma were defined as subjects with secondary recurrence.

### 2.2 Statistical analysis

Patient characteristics were summarized as frequencies (percentage), means (standard deviation [SD]), or medians (interquartile range). For categorical variables, differences between groups were estimated by Fisher exact test. For continuous variables, differences between groups were estimated by Wilcoxon rank-sum test.

OS was defined as the time from date of first confirmed recurrence to time of death, last visit, or December 2015, and PFS was defined as the time from date of first recurrence to date of secondary recurrence based on clinical evidence or end of the study period. Disease progression was determined by MRI based on response assessment in neuro-oncology criteria.^[5]^ In the current study, 60-month (5-year) OS and PFS rates were determined. OS and PFS were reported with 95% confidence intervals (CIs). Survival curves were determined using the Kaplan–Meier method, and differences were estimated by the log-rank test. Univariate analysis of treatment response for all recurrent glioblastoma patients and secondary recurrence patients in association with parameters were evaluated using Wilcoxon rank-sum test or the Kruskal–Wallis test. *P* values less than .05 were considered statistically significant. All statistical analyses were performed using Stata (StataCorp. 2009. Stata 11 Base Reference Manual. College Station, TX: Stata Press).

## 3 Results

### 3.1 Demographic and clinicopathologic characteristics

Among a total of 22 patients who fulfilled the inclusion criteria of the study, there were 7 and 15 patients in the chemotherapy-negative and chemotherapy-positive groups, respectively. There were no significant differences in baseline patient characteristics including age at diagnosis, sex, or Karnofsky performance status score distribution between the 2 groups (Table 1). Patients in the chemotherapy-positive group were younger than those in the chemotherapy-negative group (median, 49.5 vs 56.3 years). The proportion of females was higher in the chemotherapy-negative group than in the chemotherapy-positive group (85.7% vs 33.3%); this difference between the 2 groups, while significant by one-sided Fisher exact test (*P* = .032), failed to be significant by two-sided Fisher exact test (*P* = .067). All primary recurrences in the chemotherapy-negative group were in non-eloquent areas, whereas only 80% of the primary recurrences in the chemotherapy-positive group were in non-eloquent areas, with the remaining 20% of the primary recurrences occurring in eloquent areas, no significant different between 2 groups (*P* = .523). Main type of recurrence was local in the chemotherapy-positive group (71.4%), which was observed in 66.7% of the chemotherapy-negative patients, and no significant different between 2 groups (*P* = 1.000). The proportion of patients with a Karnofsky performance status score <60 was higher in the chemotherapy-positive group than in the chemotherapy-negative (60.0% vs 42.9%), but no significant different between 2 groups (*P* = .652). Second surgery after recurrence was performed in 71.4% and 80.0% (*P* = .829) of the patients in the chemotherapy-negative and chemotherapy-positive groups, respectively.

**Table 1:**
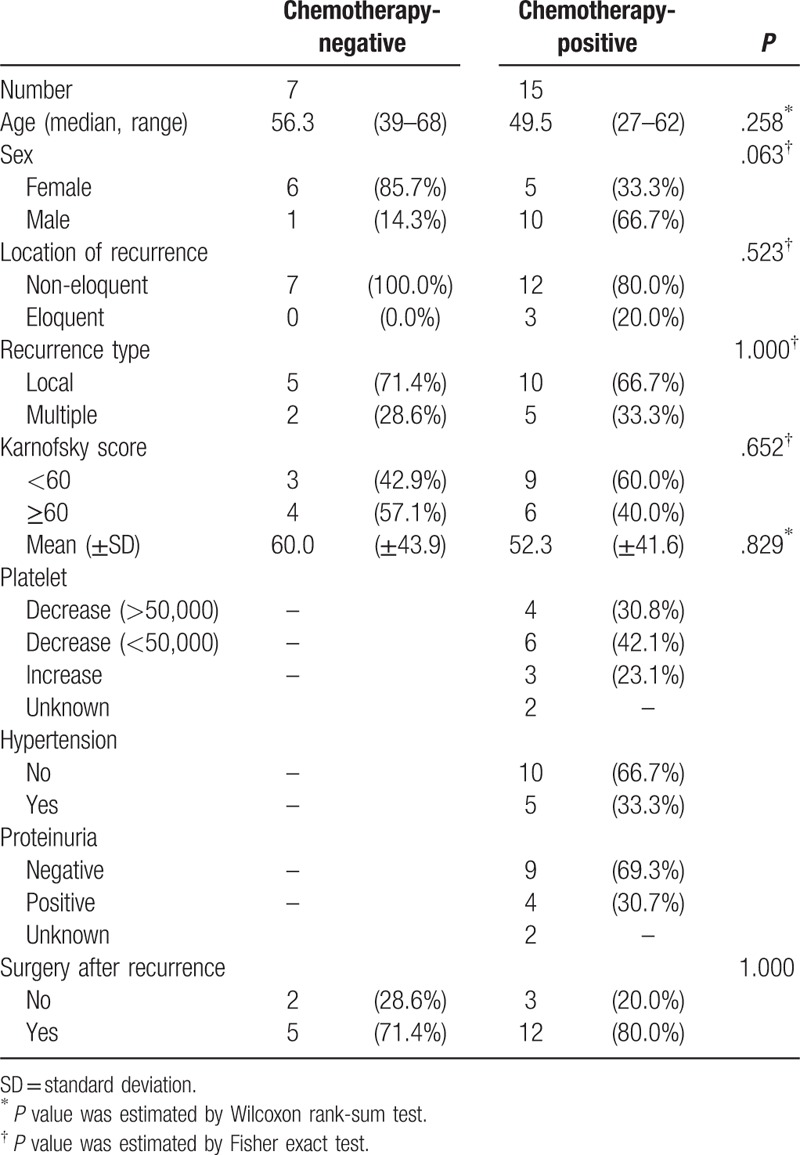
Baseline characteristics (n = 22).

Data on platelet counts, presence of hypertension, and proteinuria were available only for patients in the chemotherapy-positive group. In this group, the reduction in platelet count was less than 50,000/mm^3^ after chemotherapy in 30.8% of the patients, which was more than 50,000/mm^3^ in 42.1% of the patients; there was an increase in platelet counts in only 23.1% of the patients after chemotherapy treatment. Additionally, the rates of hypertension and proteinuria were 33.3% and 30.7%, respectively, among patients in the chemotherapy-positive group.

**Figure 1:**
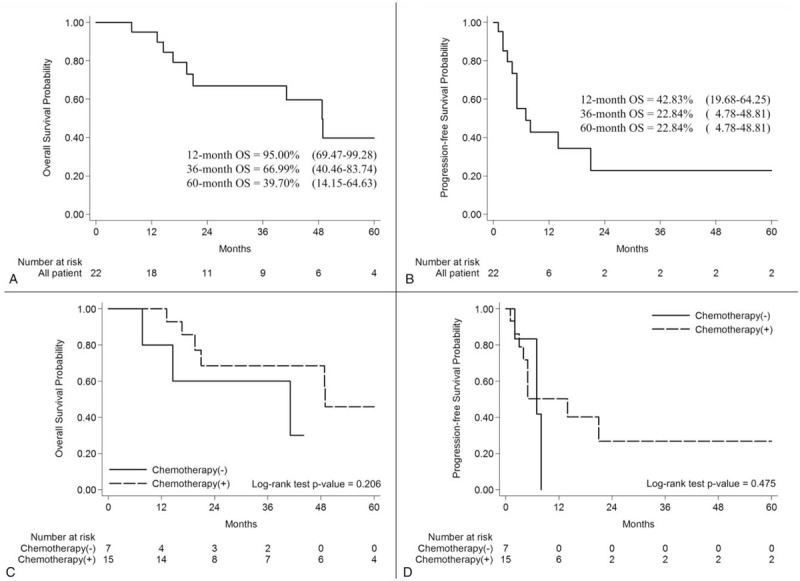
(A) Overall survival of all patients. (B) Progression-free survival of all patients. (C) Overall survival for patients receiving bevacizumab in combination with vincristine and low-dose carboplatin and those not receiving chemotherapy. (D) Progression-free for patients receiving bevacizumab in combination with vincristine and low-dose carboplatin and those not receiving chemotherapy. Chemotherapy(–) represents chemotherapy-negative group. Chemotherapy(+) represents chemotherapy-positive group.

A total of 6 (27.3%) of the 22 patients died within the follow-up duration in this study. Median OS in the entire cohort was 10.0 (0.7–89.3) months. In the entire cohort, 12-, 36-, and 60-month OS rates were 95.00% (95%CI, 69.47%–99.28%), 66.99% (95%CI, 40.46%–83.74%), and 39.70% (95%CI, 14.15%–64.63%), respectively (Fig. 1A). Secondary recurrence occurred in 12 (54.6%) patients at a median of 4.5 (0.7–21.0) months. Finally, PFS rate for secondary recurrence in 12-month was 42.83% (95%CI, 19.68%–64.25%), and 22.84% (95%CI, 4.78%–48.81%) for both 36- and 60-months (Fig. 1B).

### 3.2 Treatment response

Mortality rates were similar between the chemotherapy-negative and chemotherapy-positive groups (3/7 [42.86%] vs 6/15 [40.0%], one-sided Fisher exact test *P* value = .628). However, the secondary recurrence rate was higher in the chemotherapy-positive group (9/15, 60.0%) than the chemotherapy-negative group (3/7, 42.9%), but no significant different was found (one-sided Fisher exact test *P* value = .384). Table 2 summarizes median OS and PFS rates based on treatment approaches in patients with recurrent glioblastoma. Patients in the chemotherapy-positive group exhibited a significantly longer median OS compared to those in the chemotherapy-negative group (*P* = .006), with median OS as 13.5 (6.5–89.3) and 3.2 (0.7–14.8) months in the chemotherapy-positive and chemotherapy-negative groups, respectively. As shown in Figure 1C, a similar trend in OS rates was observed between the chemotherapy-positive and chemotherapy-negative groups, but no statistical significance was found by the log-rank test (*P* = .206). Median PFS of the chemotherapy-positive group (5.0 [1.0–21.0] months) was also longer than that of the chemotherapy-negative group (2.7 [0.7–8.0] months). Although there was no significant difference in PFS rates between the 2 groups (*P* = .475; Fig. 1D), the PFS curve of the chemotherapy-positive group was slightly better than that of the chemotherapy-negative group. This finding suggested that patients with recurrent glioblastoma treated with the combination chemotherapy might achieve a comparable stable disease status after 6 months of follow-up.

**Table 2:**
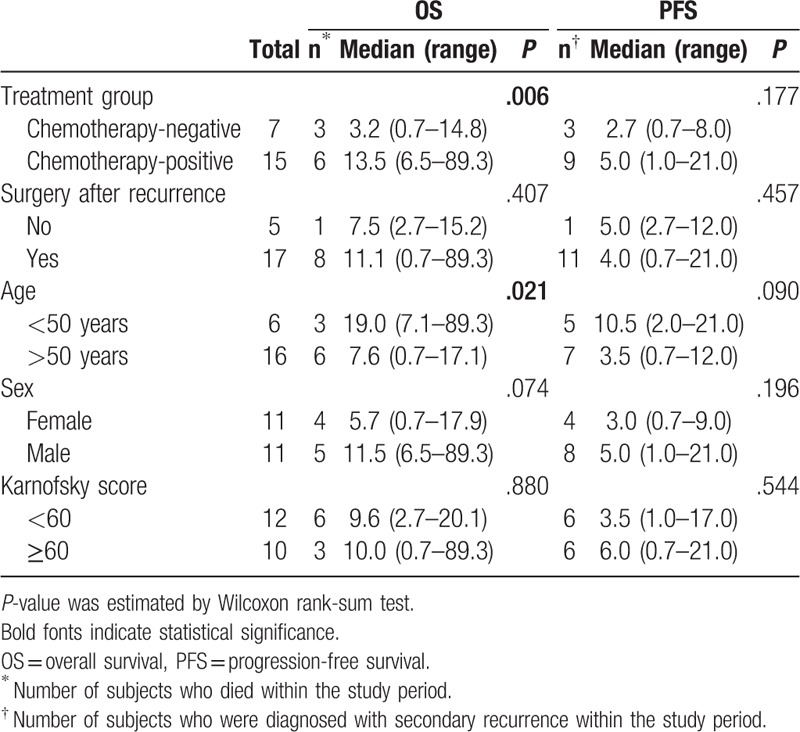
Treatment efficacy.

The median OS was longer in patients who underwent surgery after recurrence (11.1 [0.7–89.3] months) than those who did not undergo surgery (7.5 [2.7–15.2] months). However, surgery was not associated with a benefit in PFS. The median PFS was shorter in patients who underwent surgery after recurrence (4.0 [0.7 and 21.0 months) than those who did not undergo surgery after recurrence (5.0 [2.7–12.0] months). Median OS was significantly longer in patients who were younger than 50 years (19.0, [7.1–89.3] months) than in those who were 50 years or older (7.6, [0.7–17.1] months). Similar results were found for PFS; however, there was no significant difference in PFS rates between the 2 groups based on different age groups. There were no significant associations between sex and Karnofsky performance status score and median OS or PFS.

### 3.3 Response of treatment based on clinical imaging findings

**Figure 2:**
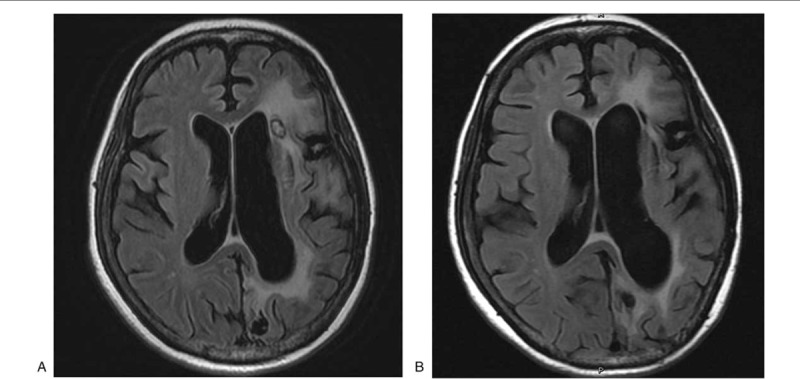
(A) MRI scan of a patient at the time of diagnosis of initial recurrence of glioblastoma. (B) Post-chemotherapy MRI scan of the same patient showing obvious shrinkage of tumor mass. MRI = magnetic resonance imaging.

Comparison of MRI findings at the time of diagnosis of initial recurrence and post-chemotherapy is presented in Figure 2. The benefit of chemotherapy was evident in decreases in tumor mass and perifocal edema. An obvious shrinkage of tumor mass was noted in follow-up MRI. The patient demonstrated partial response after receiving bevacizumab/vincristine/carboplatin combination chemotherapy.

### 3.4 Prognostic factors associated with secondary recurrence in patients receiving bevacizumab/vincristine/carboplatin combination chemotherapy

As presented in Table 3, none of the variables was found to be a significant prognostic factor; however, patients with better median OS and PFS months following bevacizumab/vincristine/carboplatin combination chemotherapy exhibited several characteristics including a decrease in the platelet count following chemotherapy, chemotherapy-related hypertension, and acceptable proteinuria.

**Table 3:**
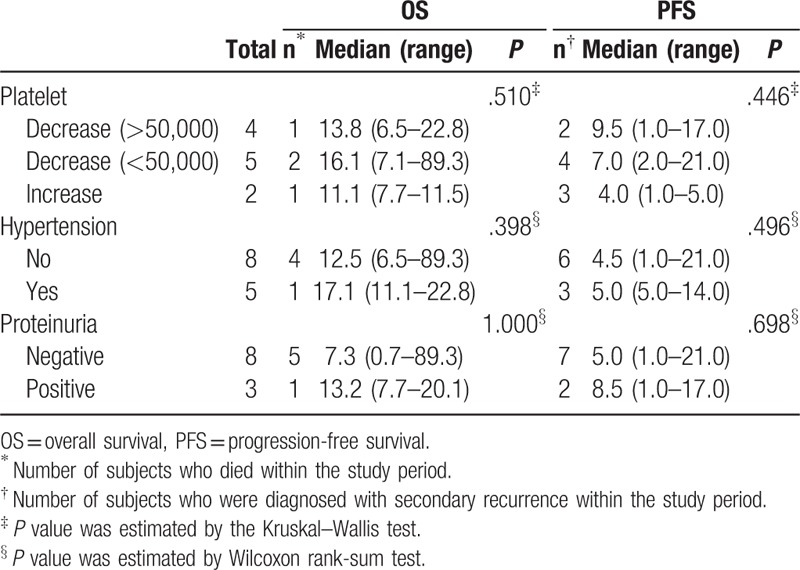
Prognostic factors for overall survival and secondary recurrence in patients treated with bevacizumab combination chemotherapy.

## 4 Discussion

Previous studies have shown that combination chemotherapy regimens including bevacizumab (bevacizumab plus valganciclovir or bevacizumab plus lomustine) exhibited only modest activity in patients with recurrent glioblastoma. However, the findings of the current study demonstrated that survival was improved with bevacizumab/vincristine/carboplatin combination chemotherapy in this patient population.

Standard treatment for newly diagnosed glioblastoma is extensive tumor resection followed by the Stupp treatment protocol^[32]^; however, there are currently no established treatment protocols for recurrent glioblastoma. Due to their highly vascularized nature, antiangiogenic agents are widely used in recurrent forms of glioblastoma. OS and PFS rates of recurrent glioblastoma were found not to have improved despite bevacizumab chemotherapy in a recent systematic review,^[33]^ whereas combination chemotherapy protocols including bevacizumab appear to achieve better clinical outcomes.^[14]^

In addition, a nonsignificant age difference was found either in the same group as well as between groups. Although age difference is considered as a prognosis factors for many diseases, the impact of age in glioblastoma still insufficiently reported. Previous studies on generally indicated the prognosis prediction for glioblastoma were more likely associated with biomarker and pathological factors.^[34,35]^ Furthermore, the small sample size of current study was difficult to conduct a few subgroup analyses for age stratification. Hence, we have divided the patients in 2 groups including age <50 years and >50 years in order to reduce the influence of age different in OS and PFS estimation. The results indicated the patients with age <50 years have significantly longer OS interval compared to the elder group.

Bevacizumab, vincristine, and carboplatin were used concurrently to treat glioblastoma patients in the current study, based on their distinctive mechanisms of action. The results demonstrated that bevacizumab/vincristine/carboplatin combination chemotherapy might potentially prolong OS. Although a significant benefit was not found in secondary PFS, the findings suggest that bevacizumab/vincristine/carboplatin combination chemotherapy might potentially promote stable disease status for recurrent glioblastoma after 6 months of follow-up (Fig. 1D). Contrary to the findings of previous studies, repeat surgery after recurrence was not found to be associated with a benefit in OS or PFS.^[36]^ Furthermore, our analysis revealed that clinical outcomes were better after bevacizumab/vincristine/carboplatin combination chemotherapy in younger patients. Finally, there were slight improvements in tumor control and OS in patients who developed bevacizumab-induced hypertension and proteinuria and those whose platelet counts were decreased.

Before the introduction of bevacizumab, concurrent vincristine and carboplatin use was associated with exceptional treatment results in glioblastoma.^[23,26]^ Vincristine, a microtubule-destabilizing drug that exhibits antiangiogenic and antitumoral activity, was shown to affect VEGF expression in glioblastoma cells.^[22]^ Conversely, carboplatin kills tumor cells by interfering with deoxyribonucleic acid (DNA) duplication^[37,38]^ and is usually used in patients with worst disease status due to its severe toxicity characteristics. Based on in-depth investigation of glioblastoma and improved understanding of basic mechanisms of tumorigenesis, bevacizumab was proposed for treatment of glioblastoma based on its inhibitory action on upstream mediators of tumor angiogenesis. A recent study demonstrated the significant impact of bevacizumab-including combination treatments on transcriptional changes in glioblastoma.^[39]^

Common serious side effects of bevacizumab are gastrointestinal perforation, serious bleeding, proteinuria, hypertension, and poor wound healing. The current study did not find any bevacizumab-related serious complications such as gastrointestinal perforation, serious bleeding, or new-onset seizure during chemotherapy. Other side effects such as proteinuria and hypertension were more common and well controlled by medication. Due to the use of low-dose carboplatin, no additional severe side effects were observed with combination chemotherapy in the current study. Bevacizumab, vincristine, and carboplatin are included in the National Comprehensive Cancer Network guidelines for glioblastoma treatment due to their inhibitory actions on distinct pathways. While the response of the combinational use of these therapeutics was seldom reported, we hypothesize that their combination use might exert a possible synergistic effect via inhibition of the interaction between VEGF and VEGFR, inhibition of VEGF expression in glioblastoma cells, and tumor cell death by interruption of DNA duplication, all in the context of low toxicity.

Although the retrospective study design limited additional data collection and analysis for the primary outcome, this single-center, single-physician design likely minimized the selection and information bias. However, the retrospective design might have hindered control of confounders which were reported previously.^[40–43]^ Due to the small sample size in the current study, we could not perform the Cox proportional hazards model to estimate OS and PFS based on specific baseline characteristics and clinicopathologic factors. It is also undeniable that the small sizes might reduce the generalizability of the current study results. Recent studies demonstrated the contribution of genetic factors in glioblastoma treatment, and new discoveries in genomics are likely to bring breakthrough findings to benefit treatment outcomes.^[44–46]^ Previous studies indicate that targeted inhibitors against the MET oncogene might be another therapeutic option for pediatric glioblastoma patients expressing a MET fusion protein,^[44]^ and another in vivo study demonstrated that the transcriptional inhibitor mithramycin could reduce proliferation of glioblastoma cells by downregulating SOX2 and its target genes.^[46]^ Alternative genetic therapeutic strategies such as targeted inhibitors against specific oncogenes or downregulation of specific cell proliferation pathways, together with bevacizumab/vincristine/carboplatin combination chemotherapy treatment require further experimental and clinical proof for efficacy and clinical translation.

This is the first study to report the treatment response of bevacizumab in combination with vincristine and low-dose carboplatin in recurrent glioblastoma in a Taiwanese cohort. For recurrent glioblastoma or patients who have no advantaged in primary surgery, the study results suggested the combination regimen of bevacizumab, vincristine, and low-dose carboplatin could be considered as a viable therapeutic approach. In addition, the patients who aged under 50 years were more likely to obtain longer survival interval. Along with advances in oncology, more in-depth understanding of the mechanism underlying this combination treatment in recurrent glioblastoma is necessary.

## 5 Declarations

### 5.1 Ethics approval and consent to participate

This study was approved by the institutional review board (IRB) of Kaohsiung Medical University hospital (IRB number: KMUHIRB-G(II)-20170010). Written informed consent was obtained from the patient for clinical data collection including medical records and/or clinical images.

### 5.2 Consent to publish

Not applicable.

### 5.3 Availability of data and materials

The data that support the findings of this study are available from Kaohsiung Medical University Hospital but restrictions apply to the availability of these data, which were used under license for the current study, and so are not publicly available. Data are however available from the authors upon reasonable request and with permission of Kaohsiung Medical University Hospital.

## Author contributions

**Conceptualization:** Yu-Kai Huang, Ann-Shung Lieu.

**Data curation:** Yu-Kai Huang, Ann-Shung Lieu.

**Formal analysis:** Yu-Kai Huang.

**Funding acquisition:** Ann-Shung Lieu.

**Methodology:** Yu-Kai Huang.

**Project administration:** Ann-Shung Lieu.

**Supervision:** Ann-Shung Lieu.

**Visualization:** Yu-Kai Huang.

**Writing – original draft:** Yu-Kai Huang.

**Writing – review & editing:** Ann-Shung Lieu.

Ann-shung Lieu orcid: 0000-0002-6953-0662.

## Supplementary Material

Supplemental Digital Content
