# Supplementary material for: Treatment response of bevacizumab combination chemotherapy in recurrent glioblastoma: A long-term retrospective study in Taiwan
Source: Medicine (Baltimore). 2020 Feb 21;99(8):e19226. doi: 10.1097/MD.0000000000019226 (PMC7034747; doi:10.1097/MD.0000000000019226)

**Appendices**

**APPENDIX A**. Treatment flowchart for glioblastoma patients. Chemotherapy(-) represents chemotherapy-negative group. Chemotherapy(-) represents chemotherapy-positive group.


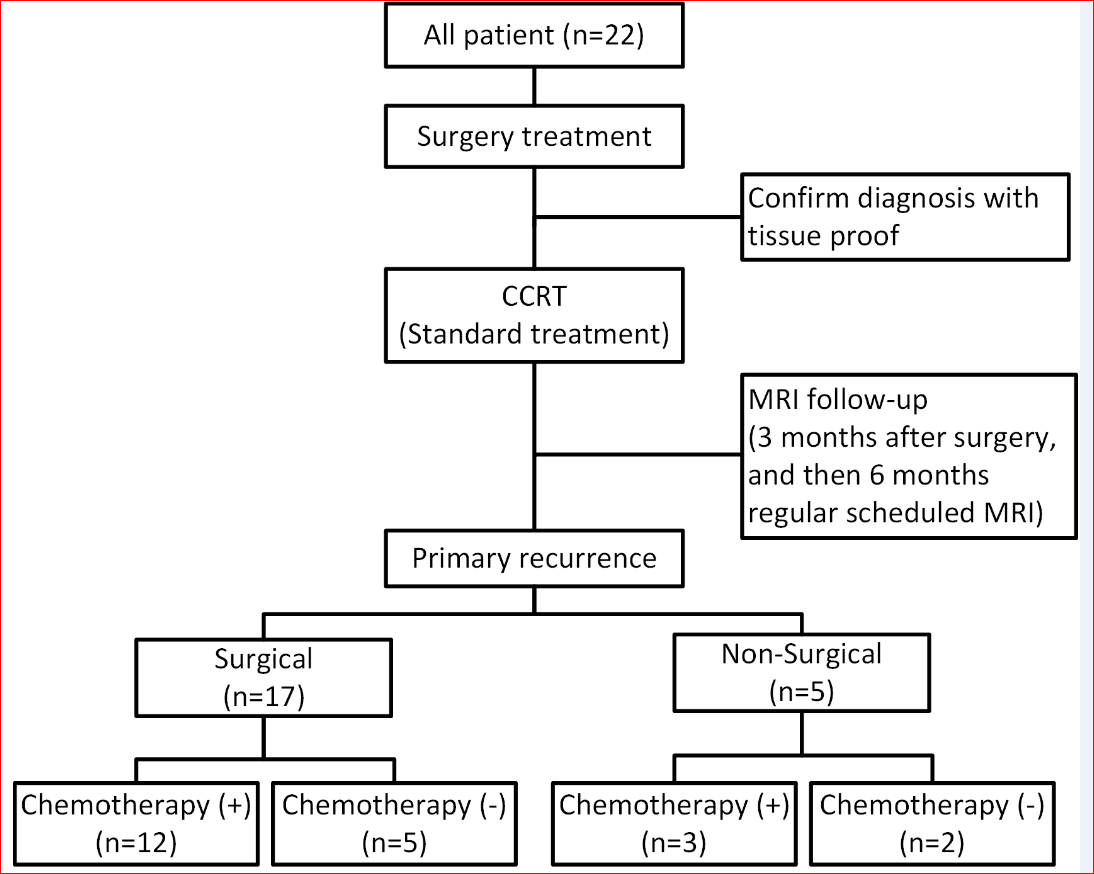

Supplement: Supplemental Digital Content [file medi-99-e19226-s001.doc]
